# Fetal profile in fetuses with open spina bifida

**DOI:** 10.1007/s00404-020-05503-8

**Published:** 2020-03-24

**Authors:** Natalia Prodan, Markus Hoopmann, Jiri Sonek, Christoph Oettling, Harald Abele, Philipp Wagner, Karl Oliver Kagan

**Affiliations:** 1 Department of Obstetrics and Gynecology, University of Tuebingen, Calwerstrasse 7, 72076 Tübingen, Germany; 2 Fetal Medicine Foundation USA, Dayton, OH USA; 3 Division of Maternal Fetal Medicine, Wright State University, Dayton, OH USA

**Keywords:** Spina bifida, Profile, Prefrontal space ratio, Frontomaxillary angle

## Abstract

**Objective:**

To determine whether the frontomaxillary facial (FMF) angle and the prefrontal space ratio (PFSR) are helpful in screening for open spinal defects by ultrasound in the second and third trimesters of pregnancy.

**Methods:**

The FMF angle and the PFSR were measured in fetuses with spina bifida according to standardized protocols. The normal range of the PFSR was previously published by our group. To determine the normal values for the FMF angle in the second and third trimesters of pregnancy, we used the same stored images from the above-mentioned study.

**Results:**

71 affected and 279 normal fetuses were included in this study. Median gestational ages in the two groups were 21.1 weeks and 21.6 weeks, respectively. In fetuses with spina bifida, the FMF angle was significantly smaller than in the normal population (72.9° versus 79.6°). However, the measurement was below the fifth centile in only 22.5% of the affected fetuses. The PFSR was similar in both groups.

**Conclusions:**

The FMF angle is smaller in second and third trimester fetuses with open spina bifida. However, the difference is not large enough to implement this marker in current screening programs.

## Introduction

Spinal dysraphism is among the most frequent and severe congenital anomalies encountered. It includes open and closed spina bifida. According to the Eurocat registry, the prevalence is about 1 in 1000 and has not changed, despite folic acid supplementation [1, 2]. In Europe, about 90% of the pregnancies are terminated after the prenatal detection of an open spine defect [3]. These figures may change as the option of antenatal repair is becoming more available. However, it is likely that a large proportion of these couples will still decide on the termination of pregnancy in the future [4].

Open spina bifida is characterized by an exposure of nervous tissue through a defect in the spine and skin, which is due to an inadequate closure of the primary neural tube [2]. These defects lead to a variable amount of neurologic deficit. In the great majority of cases, open spine defects are associated with type II Chiari malformation. As a result, ultrasound examination of the posterior fossa along with the search for other indirect cerebral signs, such as ventriculomegaly and frontal scalloping, allows for a significantly improved detection rate of spina bifida in the second trimester of pregnancy [5, 6]. However, none of these ultrasound markers achieved a 100% detection rate [5].

Efforts have been made to improve the detection of this malformation in the first trimester. Similar to the second trimester, most studies in the first trimester focus on the posterior fossa [7, 8]. It appears that open spinal defects are associated with a caudal displacement of the midbrain and subsequent collapse of the fourth ventricle. In the first trimester, sonographically this translates into an increased ratio between the brainstem thickness and the distance between the brainstem and the occipital bone [9]. Another consequence of the brain displacement is the decrease of the fetal frontomaxillary facial (FMF) angle [10]. Lachmann et al. have shown that this observation may also be useful in early screening for spina bifida [11].

In the second trimester, the facial profile has been examined extensively. Several study groups have focussed on a standardized assessment, especially with the measurement of the FMF angle and the prefrontal space ratio (PFSR). For example, in about 80% of the fetuses with trisomy 21, the facial angle was increased and the PFSR decreased [12, 13].

The aim of our study is to determine whether the facial angle and the PFSR can be helpful in detecting open spinal defects in the second and third trimesters.

## Materials and methods

This is a retrospective study utilizing stored 2D images of second and thirdtrimester fetal profiles. The prenatal ultrasound examinations used in this study were performed at the Department of Prenatal Medicine at the University of Tuebingen, Germany, between 2007 and 2017.

**Fig. 1:**
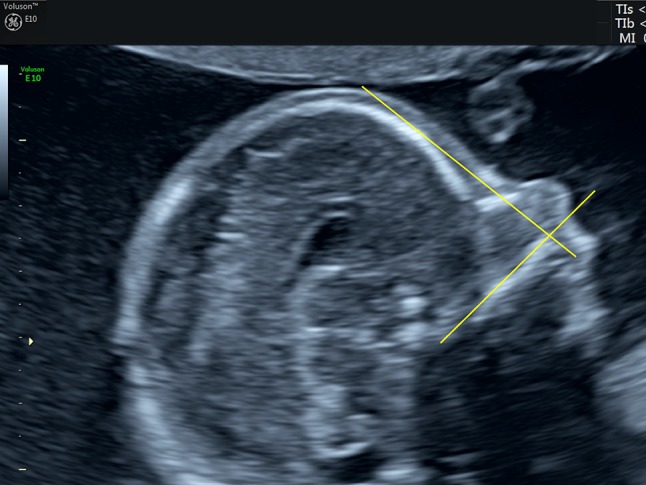
Frontomaxillary angle in a normal fetus

**Fig. 2:**
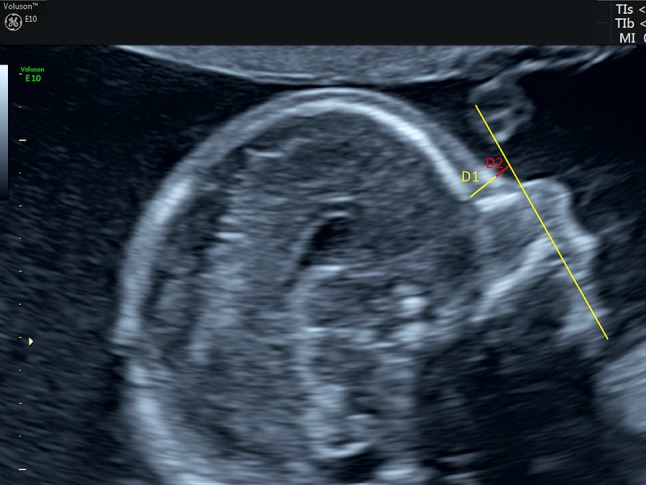
Prefrontal space ratio in a normal fetus

The measurement of the FMF angle and the PFSR were described in detail elsewhere [12–14]. In short, the FMF angle is defined as the angle between the upper surface of the palate and the frontal bone in a midsagittal view of the fetal face (Fig. 1). The PFSR is obtained by dividing the distance between the leading edge of skull and the prenasal skin (*D*1) to the distance from prenasal skin to the point where the mandibulomaxillary line is intercepted (*D*2) (Fig. 2). For an image to be acceptable for the assessment, it has to meet the following criteria: true midsagittal section (preferably with the corpus callosum visible) and clearly identifiable anterior edges of the mandible and maxilla as well as the leading edge of the bony forehead and the skin over the forehead. The magnification is such that the profile fills the majority of the image.

For the data acquisition, we searched the digital database for pregnancies in which the diagnosis of an open spinal defect had been made and that had an ultrasound examination after 14 weeks’ gestation. In pregnancies in which more than one examination was performed, only the images from the earliest suitable examination were used in our analysis***.*** Two operators (N.P. and C.O.) obtained the measurements on which the PFSR was based as well as the measurements of the FMF angle. Both were blinded to their own results and the results of the other operator.

The normal range for the PFSR was previously published by our group and was based on the assessment of 279 normal controls [13]. These measurements were not repeated for the purpose of the current study. To calculate the normal range for the FMF angle, we used the same stored images from [13].

The following data were recorded in each case: maternal history, gestational age, and fetal head biometry. In the affected cases, we also recorded the level of the spinal defect.

This retrospective study was approved by the ethical committee of the University of Tuebingen (357/20129BO2).

### Statistical analysis

For the normal range of the FMF angle, we used regression analysis to search for significant covariates. The normal range was then computed based on gestational age. The normal range of the PFSR was already published by Yazdi et al.

In addition to the PFSR which was calculated as *D*2/*D*1, we also calculated the total *D* distance as a sum of *D*1 and *D*2 [13].

In the group of affected and unaffected fetuses, each measurement was transformed into MoM (multiple of median) values. The results of both groups are shown as median and interquartile range (IQR) compared with a Mann–Whitney *U* test. The statistical analysis was carried out with IBM SPSS 24 (Armonk, New York, USA). A *p* value of < 0.05 was set as significance threshold.

## Results

The search of the database identified 85 fetuses with open spina bifida where the fetal profile was recorded. Nine pregnancies were excluded due to additional defects including facial and chromosomal abnormalities and in five cases, the profile was not recorded in a midsagittal section. Thus, we included 71 fetuses for further analysis. In 27 and 29 cases, the spinal defect started at the lumbar and sacral level, respectively. In 15 cases, the defect was either in the thoracic or cervical region. The control group consisted of 279 normal fetuses. Median gestational age in the normal and the affected groups was 21.1 and 21.6 weeks, respectively. The maternal and pregnancy characteristics are summarized in Table 1.

**Table 1:** Characteristics of the study population

	Normal (*n* = 279)	Spina bifida (*n* = 71)
Maternal age in years Median (IQR)	30.5 (19.0–45.9)	31.9 (28.0–35.0)
Gestational age in weeks Median (IQR)	21.1 (15.0–40.0)	21.6 (20.1–25.1)
Second trimester US *n* (%)	211 (75.6)	56 (80.0)
Third trimester US *n* (%)	68 (24.4)	14 (20.0)
Body mass index in kg/m^2^ Median (IQR)	25.1 (22.0–27.5)	26.2 (22.0–27.5)

### Frontomaxillary (*FMF)* angle

**Fig. 3:**
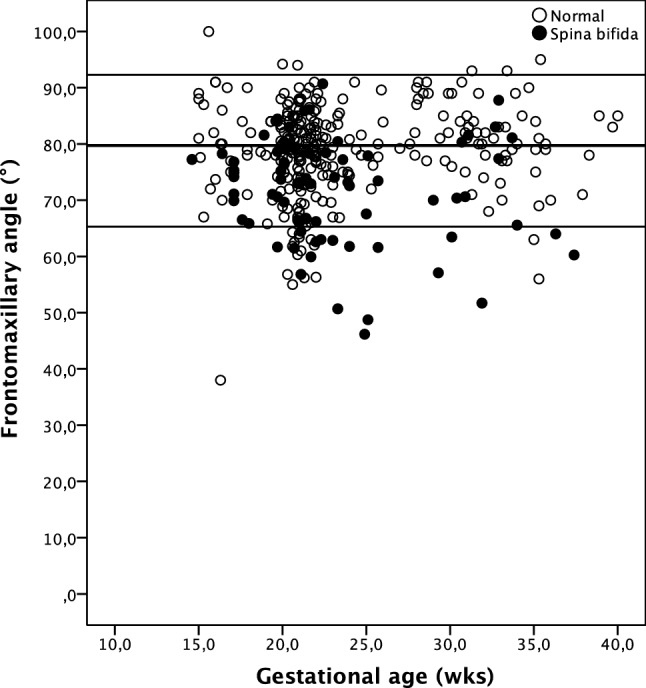
Frontomaxillary angle in normal fetuses and in fetuses with open spina bifida

In the normal group, the median frontomaxillary angle was 79.1° irrespective of the gestational age (*r* = 0.002; *p* = 0.098), head circumference (*r* = 0.013; *p* = 0.826), and biparietal diameter (*r* = 0.099; *p* = 0.975). In the spina bifida group, the median frontomaxillary angle was 72.9°. This was significantly smaller than in the normal population (*p* < 0.0001) (Fig. 3; Table 2). The angle was independent of gestational age (*p* = 0.265) in the spina bifida group as well. The angle was also not affected by the size of the defect (*p* = 0.340) and its level (using sacral defect as reference: lumbar defect *p* = 0.367, thoracic and cervical defect *p* = 0.288). In 16 (22.5%) of the affected cases, the measurement was at or below the 5th centile of the normal population.

**Table 2:** Frontomaxillary angle and prefrontal space ratio in normal fetuses and fetuses with open spina bifida

	Normal	Spina bifida
Frontomaxillary angle in °		
Absolute median (IQR)	79.6 (74.4–84.0)	72.9 (64.5–78.4)*
MoM median (IQR)	1.00 (0.93–1.06)	0.92 (0.81–0.99)
Prefrontal Space Ratio		
Absolute median (IQR)	0.95 (0.79–1.13)	0.85 (0.66–1.18)**
MoM median (IQR)	0.98 (0.82–1.17)	0.88 (0.68–1.22)
*D*1 distance in mm		
Absolute median (IQR)	4.30 (3.70–5.20)	4.35 (3.41–5.41)
MoM median (IQR)	.99 (0.91–1.09)	1.02 (0.91–1.14)***
*D*2 distance in mm		
Absolute median (IQR)	4.30 (3.20–5.20)	3.75 (2.81–5.43)
MoM median (IQR)	0.97 (0.80–1.17)	0.90 (0.67–1.20)****
Total *D* distance		
Absolute median (IQR)	8.60 (7.20–10.20)	8.00 (6.61–10.71)
MoM median (IQR)	0.99 (0.88–1.11)	0.96 (0.83–1.11)*****

Mann–Whitney *U* test *p = < 0.0001; **p = 0.066; ***p = 0.428; ****p = 0.351; *****p = 0.233

### Prefrontal space ratio

**Fig. 4:**
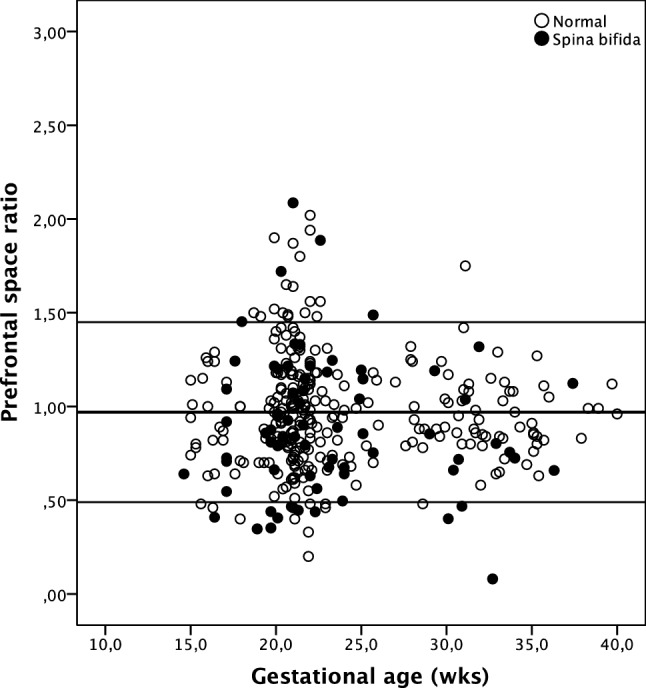
Prefrontal space ratio in normal fetuses and in fetuses with open spina bifida

In the normal group, median *D*1 and *D*2 distances and the total *D* distance were 4.30, 4.30, and 8.60 mm, respectively. Median PFSR was 0.95 (Fig. 4; Table 2). The PFSR was independent of gestational age (*r* = 0.030; *p* = 0.619), but there was a significant correlation with the head circumference (*r* = 0.140; *p* = 0.020) and on the biparietal diameter (*r* = 0.128; *p* = 0.034). As expected, *D*1, *D*2, and total *D* distance increased with advancing gestational age (PFSR *p* = 0.632, *D*1 = −0.275 + 0.205 × gestational age, *p* < 0.0001, *r* = 0.866; *D*2 = −0.079 + 0.188 × gestational age, *p* < 0.0001, *r* = 0.649; total *D* = −0.275 + 0.205 × gestational age, *p* < 0.0001, *r* = 0.866). Based on these normal ranges, median MoM value for the *D*1, *D*2, and the total *D* measurements was 0.99, 0.97, and 0.99, respectively. PFSR was similar in the normal and the spina bifida cohorts and as were the gestational age-adjusted *D*1, *D*2, and total *D* measurements (Table 2).

## Discussion

In this study, we have compared the FMF angle and the PFSR in fetuses with and without spina bifida. In the normal population, the FMF angle was similar to previous studies [15, 16]. In fetuses with spina bifida, the FMF angle was significantly smaller than in the normal population. However, the measurement was below the 5th centile only in about a fifth of the affected fetuses. The PFSR was similar in both groups. As such, we do not believe that assessment of the profile improves the detection of fetuses with open spina bifida.

A timely diagnosis of spina bifida in the first and second trimester of pregnancy is essential for the appropriate counseling of parents and for the management of these pregnancies. An unfavorable fetal lie may hinder the direct recognition of the spinal defect and this makes the diagnosis especially challenging. In these situations, the sonographer must rely almost exclusively on indirect ultrasound signs. Most fetal surrogate markers for spina bifida are found in the posterior fossa. Open spinal defects lead to a downward displacement of the hindbrain, which results in a Chiari type II malformation of the cerebellum [6]. On ultrasound, this translates into a decreased transcerebellar diameter, a misshapen cerebellum (“banana” shape), and obliteration of the cisterna magna. Further, second trimester signs of open spina bifida are a small biparietal diameter or head circumference, ventriculomegaly, and frontal bone scalloping (“lemon” shape) [5].

Although these markers do improve the detection rate of open spina bifida in the second trimester and are almost instantly recognizable by an experienced sonographer, they still do not enable a diagnosis in all cases. Ghi et al. reviewed the prenatal detection rate for open spina bifida in the Emilia-Romagna region between 2001 and 2011. The overall detection was about 80% and the diagnosis was made prior to 23 weeks in only 73% of the cases [17]. However, in a retrospective evaluation of 627 fetuses, Bahlmann et al. found a high association between posterior fossa abnormalities and open spine defects at 18–22 weeks’ gestation [5]. In euploid and aneuploid fetuses, the Chiari II malformation was present in 97% and 95% of the cases, respectively.

More recent studies have focused on first trimester detection of spinal defects. Sepulveda et al. reviewed the current body of literature and demonstrated that all relevant first trimester ultrasound markers result from the hindbrain displacement [18]. In a screening study in Berlin, the test performance of these markers was examined prospectively. With the combined use of all markers, all affected fetuses were either detected or suspected at 11–13 weeks’ gestation [7]. Lachmann et al. looked for associated changes in the shape of the fetal profile in the first trimester by measuring the FMF angle [11]. The rationale for this approach lies in the backward tilting of the forehead because of hindbrain displacement. They found that the facial angle was about 10° lower in fetuses with an open spine defect than in the normal population and in about 90% of the cases, the angle was below the 5th centile. Our study demonstrates that this finding is also present in the second and third trimesters, but the difference is less pronounced.

Some limitations of our research stem from its retrospective, single-centered nature. Only those cases where a qualitatively good midsagittal profile was obtained were included in the analysis. As such, we acknowledge that if measurements are performed on inappropriate images, the detection rate may be lower.

In conclusion, we have shown that the frontomaxillary angle is smaller in second and third trimester fetuses with an open spina bifida. However, the difference is not pronounced enough to implement this marker in current screening programs for open spina bifida.
